# A randomised, multi-centre trial of total ankle replacement versus ankle arthrodesis in the treatment of patients with end stage ankle osteoarthritis (TARVA): statistical analysis plan

**DOI:** 10.1186/s13063-019-3973-4

**Published:** 2020-02-18

**Authors:** Patrick Muller, Simon S. Skene, Kashfia Chowdhury, Suzie Cro, Andrew J. Goldberg, Caroline J. Doré, on behalf of the TARVA Study Group, Stephen Bendall, Andrew Bing, Chris Blundell, Rick Brown, Clifford Butcher, Michael Butler, Tim Clough, Paul Cooke, Nick Cullen, James Davenport, Mark Davies, Sunil Dhar, Andy Goldberg, Paul Halliwell, Bill Harries, Stephen Hepple, Raj Kakwani, Mike Karski, David Loveday, Nilesh Makwana, Steve Milner, Viren Mishra, Andrew Molloy, An Murty, Martin Raglan, Benedict Rogers, Mark Rogers, Malik Siddique, Dishan Singh, Rob Smith, Rhys Thomas, Paulo Torres, Dave Townshend, Matt Welck, Ian Winson

**Affiliations:** 1 Comprehensive Clinical Trials Unit, Institute of Clinical Trials and Methodology, University College London, London, UK; 2 Department of Clinical and Experimental Medicine, School of Biosciences and Medicine, University of Surrey, Guildford, Surrey UK; 3 Imperial Clinical Trials Unit, School of Public Health, Imperial College London, London, UK; 4 UCL Institute of Orthopaedics and Musculoskeletal Science (IOMS), Royal National Orthopaedic Hospital (RNOH), London, UK

**Keywords:** Statistical analysis plan, Arthritis, Osteoarthritis, Surgery, Total ankle replacement, Arthrodesis, Randomised controlled trial, Pain-free walking, MOXFQ

## Abstract

**Background:**

The total ankle replacement versus ankle arthrodesis (TARVA) trial aims to determine which surgical procedure confers the greatest improvement in pain-free function for patients with end-stage ankle osteoarthritis. Both procedures are effective but there has not yet been a direct comparison to establish which is superior. This article describes the statistical analysis plan for this trial as an update to the published protocol. It is written prior to the end of patient follow-up, while the outcome of the trial is still unknown.

**Design and methods:**

TARVA is a randomised, un-blinded, parallel group trial of total ankle replacement versus ankle arthrodesis. The primary outcome is the Manchester-Oxford Foot Questionnaire walking/standing domain score at 52 weeks post-surgery. Secondary outcomes include measures of pain, social interaction, physical function, quality of life, and range of motion. We describe in detail the statistical aspects of TARVA: the outcome measures, the sample size calculation, general analysis principles including treatment of missing data, the planned descriptive statistics and statistical models, and planned subgroup and sensitivity analyses.

**Discussion:**

The TARVA statistical analysis will provide comprehensive and precise information on the relative effectiveness of the two treatments. The plan will be implemented in January 2020 when follow-up for the trial is completed.

**Trial registration:**

ISRCTN registry number 60672307, ClinicalTrials.gov registration number NCT02128555. Registered 1 May 2014. Recruitment started in January 2015 and ended in January 2019.

## Background

End stage ankle osteoarthritis (OA) causes pain and chronic disability [1, 2]. It has been estimated that at least 29,000 patients with symptomatic ankle OA are referred to specialist foot and ankle surgeons each year in the UK, and that around 3,000 will choose to undergo surgery with the NHS [3]. The main surgical treatments for end-stage ankle OA are total ankle replacement (TAR) or ankle arthrodesis (fusion) [4]. Improvements in pain-free function and quality of life have been reported for both TAR and fusion, but there has never been a prospective randomised trial directly comparing the two treatments [5].

TARVA is a parallel-group, non-blinded randomised controlled trial that will compare clinical outcomes and cost-effectiveness of TAR versus ankle arthrodesis in patients with end-stage ankle Osteoarthritis (OA). Full details of the background to the trial, the interventions under study and its design are in the published trial protocol [6]. From March 2015 to January 2019, 303 patients were randomised to surgery at one of 17 surgical centres participating in the trial in England, UK.

This article describes the statistical analysis plan for the TARVA trial. Analyses will commence in February 2020 following completion of 52 weeks follow-up for the last patient, data cleaning checks, and data lock. The analysis of the primary outcome will be independently programmed from the cleaned derived dataset by a statistician who did not perform the main analysis, and in parallel by the trial statistician.

### Objectives

The primary objective of the TARVA trial is to compare TAR versus arthrodesis for:

- Improvement in self-reported pain-free function from pre-operative assessment (“pre-op”) to 52 weeks after surgery (“post-op”)

The secondary objectives are to compare TAR versus arthrodesis for:

- Improvement in self-reported pain, social interaction, physical function, quality of life (QoL), and total ankle range of motion (ROM) from pre-op to 52 weeks post-op
- Improvement in self-reported pain-free function, pain, social interaction, physical function, and QoL from pre-op to 26 weeks post-op
- Safety

## Design and methods

### Design

TARVA is a two-arm, prospective, multi-centre, parallel-group, non-blinded randomised controlled trial.

### Patient eligibility criteria

Inclusion criteria:

- Diagnosis of end-stage ankle OA
- Aged 50–85 years inclusive
- The surgeon believes the patient is suitable for both TAR and arthrodesis (having considered deformity, stability, bone quality, soft tissue envelope, and neurovascular status)
- The patient can read and understand the patient information sheet (PIS) and trial procedures
- The patient is willing and able to provide written informed consent

Exclusion criteria:

- Previous ipsilateral talonavicular, subtalar, or calcaneocuboid fusion or surgery planned within 1 year of index procedure
- More than four lower limb joints fused (including contralateral limb, but excluding PIPJ fusions)
- Unable to have either an MRI or a CT scan (e.g. severe claustrophobia or contraindication for both types of scan)
- History of local bone joint infection
- Severe osteoporosis (T score < − 2.5) with recent fracture (< 12 months)
- Any co-morbidity which, in the opinion of the investigator, is severe enough to:
  - ◦ Interfere with the patient’s ability to complete the study assessments
  - ◦ Present an unacceptable risk to the patient’s safety
- The patient is participating in another clinical trial that would materially impact on their participation in this study

Patients with end-stage OA in both ankles had only one ankle randomised and operated on as part of the TARVA trial; the other ankle was treated with usual care.

### Randomisation and blinding

The randomisation process was based on a minimisation algorithm. The algorithm gave an overall 85% chance of allocating the patient to the treatment arm which was under-represented with respect to three stratifying variables: surgeon, presence of OA in subtalar joint, and presence of OA in talonavicular joint (as determined by pre-operative MRI scan [7]). The research nurse or delegated individual logged on to the sealed envelope randomisation service and provided patient information (including on stratifying variables) and the surgical treatment to be received was supplied immediately.

Patients were allocated in a 1:1 ratio to the TAR and arthrodesis arms. It was not possible to blind patients, surgeons, radiologists, and clinical assessors for the following reasons: surgeons know what procedure they are performing, radiologists and patients will be able to identify from radiographs which procedure has taken place, and patients who receive ankle arthrodesis and their assessors will invariably know their ankle is stiff (a known consequence of arthrodesis surgery) whereas those undergoing TAR will retain motion in the ankle. To protect against allocation bias, the person recruiting the patient to the study was not aware of the allocation to be assigned prior to contacting the randomisation service.

### Trial intervention

At randomisation patients were allocated to receive either TAR or ankle arthrodesis.

For TAR, the remaining damaged cartilage is removed and the joints are resurfaced with metal implants and an intervening polyethylene liner that is either fixed or mobile to act as a gliding surface. All prostheses are CE-marked.

For ankle arthrodesis, the remaining damaged cartilage is removed from the ends of the bone and the two bones are then held together in compression using screws or plates until they join to become one (bone fusion), so that there is no longer any movement at that joint.

Full details of these interventions can be located in the trial protocol [6].

## Outcomes

### Primary outcome measure

The primary outcome measure is the absolute difference between the two treatment arms in self-reported pain-free function, as measured by the Manchester-Oxford Foot Questionnaire (MOXFQ) walking/standing domain score [8] at 52 weeks post-op. The 52-week score will be used if it was taken in a window from 48 to 56 weeks post-op.

The MOXFQ standing/walking domain score has been found to be a valid and responsive measure to evaluate all types of foot and ankle surgery [9, 10], and it has additionally been shown to be more responsive for the outcomes of foot and ankle surgery patients than generic QoL measures such as the EuroQol five-dimension quality-of-life instrument (EQ-5D) and the Short Form (36) Health Survey (SF-36) [11].

### Secondary outcome measures

The secondary outcome measures for the trial are the absolute differences between the two treatment arms in the:

- MOXFQ walking/standing domain score at 26 weeks post-op
- Self-reported pain and self-reported social interaction, as measured by the MOXFQ pain and MOXFQ social interaction domain scores at 26 weeks and 52 weeks post-op
- Physical function, as measured by the Foot and Ankle Ability Measure Activities of Daily Living (FAAM-ADL) questionnaire at 26 weeks and 52 weeks post-op
- Physical function for patients involved in sport, as measured by the FAAM-Sport score at 26 weeks and 52 weeks post-op
- QoL assessed by the validated EQ-5D (EQ-5D Index and EQ-5D Visual Analogue Scale (VAS)) at 26 weeks and 52 weeks post-op
- Total ROM (degrees plantarflexion and dorsiflexion) at 52 weeks post-op, assessed by goniometer
- Proportion of patients experiencing at least one adverse event (AE)
- Proportion of patients experiencing at least one serious adverse event (SAE)
- Proportion of patients with recorded complications (including revision surgery and reoperations other than revision)

Additional outcomes are also collected for a detailed cost and cost-effectiveness analysis of TAR versus ankle arthrodesis; however, this analysis will not be performed by the TARVA statistical team so these outcomes are not described here. Further details on the cost effectiveness analysis can be found in the trial protocol [6].

### Calculation of outcome scores

#### The Manchester-Oxford Foot Questionnaire (MOXFQ)

Responses to each MOXFQ questionnaire item consist of a five-point Likert scale ranging from no limitation (scoring 0) to maximum limitation (scoring 4). Items are grouped into three domains: walking/standing (seven items), pain (five items), and social interaction (four items). Domain scores are computed by summing the patient’s responses to each item within the domain and converting to a 0–100 metric, where higher scores represent greater severity.

If a single item within any domain is unanswered it will be imputed with the mean of the respondent’s answers to the other items within that domain. If two or more questions on any domain are unanswered the overall score for that domain will not be calculated and its value will be set to missing [12]. If the entire questionnaire has not been completed all MOXFQ domain scores for that visit will be set to missing.

#### The Foot and Ankle Ability Measure–Activities of Daily Living (FAAM-ADL)

Each of 21 items on the FAAM-ADL are scored from 4 (no difficulty) to 0 (difficulty) [13]. The overall FAAM-ADL score is then calculated by summing the responses to each item completed, dividing this by the maximum score achievable based on the number of items completed (e.g. 84 if all 21 items are completed), and then multiplying the resulting fraction by 100 to return a 0–100 metric, where higher scores indicate a higher level of physical function. If an answer for one item is missing its value will be imputed as the mode of the other items; if more than one item is missing the overall score will be set to missing.

The FAAM-Sport score provides a complementary specific assessment of ability to participate in sports based on eight questionnaire items, each also scored from 0 to 4. A 0–100 metric is then generated using the same approach as for the FAAM-ADL; higher scores indicate a higher level of ability to participate in sports. Missing items will be handled using the same approach as for the FAAM-ADL.

#### EuroQol 5D quality-of-life instrument (EQ-5D)

The EQ-5D assesses current health state across five dimensions—mobility, self-care, usual activities, pain/discomfort, and anxiety/depression—with five levels (each scored 1–5, with higher scores indicating worse health state). EQ-5D dimension scores will be converted to index scores using UK population values [14]. EQ-5D index scores range from − 1 (worse than death) and then 0 (worst health state) to 1 (best health state). The EQ-5D additionally includes a visual analogue scale (EQ VAS), which allows patients to record their overall current health status on a scale ranging from 0 (worst health state) to 100 (best health state).

If any dimension score is missing, the EQ-5D index score will be set to missing. If the entirety of one component of the questionnaire (dimension score or VAS) has not been completed the associated component score will be set to missing. If the entire questionnaire has not been completed, both the EQ-5D index score and EQ-5D VAS at that visit will be set to missing.

## Sample size

The sample size calculation for the primary outcome (change in MOXFQ walking/standing domain by 52 weeks post-op) was performed using Stata/IC version 12.1 [15]. It was based on achieving 90% power to detect the minimal clinically important difference (MCID) in the primary outcome at the 5% level of significance, accounting for expected loss to follow-up. The trial is multi-centre and the outcome plausibly varies by surgeon, so the sample size was adjusted to account for clustering; the intraclass correlation coefficient (ICC) was estimated based on previous studies, and the initially computed sample size was inflated by a factor f = 1 + (m − 1) ∗ ICC [16].

The sample size calculation was partly based on the study by Dawson et al. [9] which defined the MCID in the MOXFQ when evaluating outcomes following surgery for hallux valgus. They defined it as the mean change in MOXFQ of those patients who reported feeling at least “slightly better”, and found it to be 16, 12, and 24, respectively, for the standing/walking, pain, and social interaction domains of the MOXFQ. For this trial we determined it was important to detect a difference of 12 in the change from baseline in MOXFQ standing/walking domain between the two treatment arms; a conservative choice given the threshold for a MCID in the standing/walking domain found in the Dawson et al. study was 16. The standard deviation of the walking/standing domain of the MOXFQ was estimated as 27 [11], and loss to follow-up was estimated as 10% (attrition in similar RCTs has been 5–7% [17]).

Based on these quantities, the required sample size was estimated as 118 patients per arm. Assuming an average cluster size (m) of 14 (patients per surgeon) and an ICC of 0.03 (estimated from the median of ten previous surgical studies reporting patient-reported disease-specific measures 12 months post-surgery [18]), an inflation factor of f = 1.39 was estimated, leading to a final required sample size of 164 per arm or 328 patients total.

The assumptions of ICC = 0.03 and equal numbers of patients per surgeon in the sample size calculation [19] were reviewed by the trial statistician prior to the end of recruitment using the available data. The review indicated lower ICC and also some variability in numbers of patients per surgeon compared to the original assumptions (ICC < 0.01; average cluster size = 15, standard deviation = 9.6), resulting in increased power.

## Analysis principles

### Patient population to be included in analysis

The main analysis will be conducted on an intention-to-treat (ITT) basis; all observed outcome data from patients according to their randomised surgical procedure will be used, irrespective of type of surgery received or whether surgery was performed. Sensitivity analysis (described below) will assess the impact of missing outcome data.

Additionally, if cross-over prior to surgery does occur, a per-protocol (PP) analysis will be performed for the primary outcome that only includes data from patients who undergo surgery according to their randomised surgical procedure. The FAAM-Sport questionnaire is only completed by patients who indicate they are involved in sports. Analysis of the FAAM-Sport domain will therefore always be based on the subgroup of patients who indicate they are involved in sports at baseline, following the above principles.

### Significance levels of tests and confidence intervals

All statistical tests will use a two-sided *p* value of 0.05, unless otherwise specified. There will be no formal adjustment of *p* values for any interim analyses performed. Two-sided 95% confidence intervals will be presented for all estimates.

### Baseline comparability

Baseline characteristics will be summarised by randomised treatment arm. Categorical variables will be summarised by number and percentage in each category; continuous variables will be summarised by mean and standard deviation, or median and interquartile range, as appropriate. No statistical tests of differences in baseline characteristics between groups will be done, as any differences between treatment arms must be due to chance rather than bias.

### Adjustment for design factors

Since randomisation is stratified by surgeon and presence of OA in two adjacent joints (subtalar and talonavicular), analyses of outcomes will involve adjustment for these factors (as recommended in ICH E9, section 5.7 [20]) unless otherwise indicated. Treatment effects will then be estimated conditional on surgeon and presence of OA in the two adjacent joints.

Baseline MOXFQ walking/standing domain will also be adjusted for in primary analyses where this is the outcome. Similar adjustment will be made for all continuous secondary outcome variables where a baseline measurement is recorded.

### Follow-up and losses to follow up: missing data

Missing baseline covariate data are not anticipated since covariates must be recorded to allocate treatment.

We expect that up to 10% of patients will not provide measurements at 52 weeks post-op. Numbers and percentages of missing data at each visit (baseline, weeks 26 and weeks 52) will be tabulated by treatment group for the primary and secondary outcomes (Additional file 1: Table S2).

All observed data will be included in the primary and secondary analyses. Missing outcome data will be assumed to be missing-at-random (MAR) conditional on the observed values of all other variables included in the analysis models, and so independent of the values of the unobserved data itself. As the primary outcome is the change from baseline, patients without baseline and at least one outcome score will consequently not be included in the analysis. Their inclusion, however, would not add any information to the analysis [21].

The characteristics of patients missing 52-weeks MOXFQ data will be evaluated and a sensitivity analysis will be done to examine the impact of departures from the MAR assumption (described below).

### Statistical analyses

All analysis will be carried out using Stata version 15 (or above). The results of the analyses will be reported following the principle of the ICH E3 guidelines on the Structure and Content of Clinical Study Reports [22] and CONSORT guidelines [23].

### Recruitment and follow-up patterns

The number of patients screened for eligibility will be presented. Reasons for non-admissions into the trial will be reported in a tabular form (listed in the dummy tables in Additional file 1: Table S1).

The period of data collection, including the date of the first patient’s first visit and date of the last patient’s last visit will be described. Recruitment will be presented by year and centre. The throughput of patients from those screened, those randomised, and those assessed at each visit and included in the analysis will be summarised in a CONSORT flowchart [23]. The average time between pre-op assessment and surgery in each treatment arm will be reported. The number of patients who withdraw and are unwilling to provide follow-up will be reported by treatment arm, as will the number of missing baseline, 26-week, and 52-week CRFs (Additional file 1: Table S2). Other lower limb surgeries occurring within 12 months post-op will be reported by treatment arm, with information on the type of surgery and side of the body operated on.

### Baseline characteristics

Baseline characteristics will be summarised in a table by treatment arm. The variables to be reported in the baseline tables are listed in the dummy tables (Additional file 1: Table S3).

### Trial treatment

The number of patients undergoing their randomised surgery will be reported by treatment group. Although it is made explicit that patients cannot change surgical treatment arm once it has been randomly allocated, the clinician remains free to give alternative treatment to that specified in the protocol if it is felt to be in the best interest of the patient. Any cross-overs or other treatment deviations, as well as the number of patients who did not undergo surgery of any kind, will be specified along with reasons, as detailed in the protocol deviation log.

### Analysis methods

#### Primary analysis

A multilevel repeated measures linear regression model will be used to estimate the difference between the treatment groups in MOXFQ walking/standing domain score at 52 and 26 weeks post-op. Baseline scores will be adjusted for, so the model will return identical treatment effect estimates as a model for change from baseline to 26 or 52 weeks with the same baseline adjustment [24].

This analysis model will use all available visit data (from 26 weeks and 52 weeks) to strengthen confidence in the MAR assumption and give greater power to detect differences at individual visits.

The model for the MOXFQ walking/standing domain will include fixed effects for time (two categories, 26 weeks/52 weeks), treatment (two categories, TAR/ankle arthrodesis), treatment by time interaction, baseline MOXFQ walking/standing domain (continuous), and presence of OA in each of the two adjacent joints as determined by a pre-operative MRI scan (OA1, two categories, present/absent subtalar joint; OA2, two categories, present/absent talonavicular joint). A random patient effect will be included to take account of clustering by patient. A random surgeon effect, and an additional random surgeon by treatment coefficient, will also be included in the model to take account of clustering by surgeon and variation in the treatment effect by surgeon. This will be modelled with an unstructured covariance structure. The model will be fitted using restricted maximum likelihood estimation (REML).

The model for y_ijk_, the MOXFQ walking/standing domain value at follow-up (either 26 or 52 weeks), where i indexes the visit time, j the individual, and k the surgeon, will hence be:

1 $$ {\mathrm{y}}_{\mathrm{ijk}}={\beta}_{0\mathrm{jk}}+{\beta}_{1\mathrm{k}}\left({\mathrm{treatment}}_{\mathrm{jk}}\right)+{\beta}_2\left({\mathrm{time}}_{\mathrm{ijk}}\right)+{\beta}_3\left({\mathrm{time}}_{\mathrm{ijk}}\ast {\mathrm{treatment}}_{\mathrm{jk}}\right)+{\beta}_4\left(\mathrm{baseline}\ {\mathrm{MOXFQ}}_{\mathrm{jk}}\right)+{\beta}_5\left({\mathrm{OA}1}_{\mathrm{jk}}\right)+{\beta}_6\left({\mathrm{OA}2}_{\mathrm{jk}}\right) $$

Where,

$$ {\displaystyle \begin{array}{l}{\beta}_{0\mathrm{jk}}={\beta}_0+{\mathrm{v}}_{0\mathrm{k}}+{u}_{0\mathrm{jk}}+{\varepsilon}_{\mathrm{ijk}}\\ {}{\beta}_{1\mathrm{k}}={\beta}_1+{u}_{1\mathrm{k}}\end{array}} $$

And,

$$ {\displaystyle \begin{array}{l}{\mathrm{v}}_{0\mathrm{k}}\sim \mathrm{N}\left(0,{\sigma}_v^2\right)\\ {}{u}_{0\mathrm{jk}}\sim \mathrm{N}\left(0,{\sigma}_{u0}^2\right)\\ {}{\varepsilon}_{\mathrm{ijk}}\sim \mathrm{N}\left(0,{\sigma}^2\right)\\ {}{u}_{1\mathrm{k}}\sim \mathrm{N}\left(0,{\sigma}_{u1}^2\right)\end{array}} $$

And,

$$ {\mathrm{treatment}}_{\mathrm{jk}}=1\ \mathrm{if}\ \mathrm{treatment}\ \mathrm{is}\ \mathrm{TAR}\ \mathrm{and}\ 0\ \mathrm{if}\ \mathrm{treatment}\ \mathrm{is}\ \mathrm{ankle}\ \mathrm{arthrodesis}. $$

The primary outcome is the average difference between treatment groups at 52 weeks, estimated as *β*_1_ + *β*_3._

Heterogeneity of surgeon cluster sizes may lead to model convergence problems. Although randomisation is stratified by surgeon, if a large number of surgeons only see a very few patients there may be insufficient data to estimate the random surgeon by treatment coefficient. If the primary analysis model fails to converge, the model will be refitted after excluding the random surgeon by treatment coefficient.

The model makes assumptions about random effects distributions, correlation structure, and residuals, which will all need investigation. If any assumptions are poorly met then transformation of the change in MOXFQ walking domain score may be required.

#### Secondary analysis

##### Continuous secondary outcomes

The treatment group difference in 26-week MOXFQ walking/standing domain score will be obtained from the primary analysis model (1) as *β*_1._

Each of the following continuous secondary outcome measures will be analysed using a separate multilevel repeated measures linear regression model:

- MOXFQ pain domain score
- MOXFQ social interaction domain score
- FAAM-ADL
- FAAM-Sport (for patients involved in sport)
- EQ-5D Index
- EQ-5D VAS
- ROM dorsiflexion
- ROM plantarflexion

Similar to the primary analysis model, each model will include fixed effects for treatment, time, treatment by time interaction, baseline value of the associated score, and presence of OA in each of the two adjacent joints as determined by a pre-operative MRI scan. A random patient effect, a random surgeon effect, and a random surgeon by treatment coefficient will also be included in each of the models. If convergence problems are experienced, the approach outlined for the primary outcome will be followed.

##### Adverse events, serious adverse events, and complications

The following absolute differences in proportions will be estimated using the treatment coefficient obtained from a binomial regression model with the identity link function:

- Proportion of patients experiencing at least one AE
- Proportion of patients experiencing at least one SAE
- Proportion of patients with at least one recorded complication (any complication)
- Proportion of patients requiring revision ankle surgery
- Proportion of patients experiencing reoperation other than revision
- Proportion of patients experiencing surgical site infection

Relative risks will be obtained from a binomial regression model with the log link. If convergence is an issue a Poisson regression model with the log link and robust error estimates will alternatively be fitted to obtain relative risks.

Unadjusted treatment differences will initially be obtained for each of the event outcomes. The models will then be extended to adjust for presence of OA in each of the two adjacent joints (OA1, OA2). Due to potential sparse data in these outcomes, the models will not adjust for surgeon.

The distribution of the AEs and SAEs per patient will also be presented descriptively, but no formal analysis will be performed.

### Additional analyses

#### Subgroup analyses

An exploratory subgroup analysis will be performed to investigate whether there is any interaction between the effect of treatment and the presence of OA in each of the two adjacent joints on the primary outcome.

The fitted primary analysis model will be extended to include the interactions between treatment and presence of OA in each of the two adjacent joints. As the trial has not been powered to detect this, the analysis will have limited power and is exploratory. We would anticipate that the outcomes in TAR patients at 52 weeks are better than arthrodesis patients when there is osteoarthritis in adjacent joints.

Further exploratory subgroup analyses will be undertaken similarly to investigate whether there is any interaction between patient preference (TAR, arthrodesis, or no preference) and the effect of the treatment to which the subjects are subsequently allocated, and whether there are any interactions between treatment and age, sex, or significant mal-alignment pre-surgery (as measured on plain AP radiographs, i.e. tibiotalar angle). The fitted primary analysis model will be extended to include the interaction between treatment and the associated variable for each test.

All subgroup analyses are hypothesis generating and will not form the basis of conclusions drawn from the trial.

#### Sensitivity analyses

The robustness of the results to assumptions made about missing data in the primary outcome will be assessed. The primary analysis is only valid if the distribution of the 52-week MOXFQ scores are not different between the responses which are observed and those which are unobserved (conditional on all baseline response and covariates, treatment, and 26-week post-op response), i.e. if these data are missing at random.

Firstly, characteristics of patients missing a 52-week response will be investigated using logistic regression, with an indicator for missing data modelled on baseline covariates and the data items collected at 26 weeks post-op. Results from the model will provide contextual information regarding the missing data and, together with qualitative information gathered from the site teams, will be used to explore potential mechanisms for missing data.

Secondly, if more than 10% of patients operated on are missing 52-week MOXFQ scores (the attrition assumed in the original sample size calculation), a sensitivity analysis will be done to explore the impact of the primary outcome data being missing not at random (MNAR). A pattern mixture modelling approach will be adopted for the analysis [25]. It will explore how different the unobserved responses would have to be from the observed responses for inferences from the primary analysis to change; specifically, how extreme the departure from MAR would have to be for the *p* value to change from *p* < 0.05 to *p* ≥ 0.05 (or *p* ≥ 0.05 to *p* < 0.05).

In brief, multiple imputation will be used to produce and analyse datasets with 52-week MOXFQ imputed on the assumption that it is missing randomly conditional on the other recorded variables. The number of imputation datasets created, n, will be chosen to give a power reduction of < 1% compared to using *n* = 100 [26]. Then, the imputed 52-week MOXFQ scores will each have a number Ø added to them, and the multiple imputation primary analysis model will be run (with estimates combined using Rubin’s rules [27]). The value of Ø which causes the *p* value for the 52-week treatment effect estimate to cross the 0.05 boundary will be identified and reported. This number is interpretable as how different MOXFQ would have to be from expected amongst the patients who did not attend at 52 weeks for the analysis conclusions to change. The possibility that data are MNAR in one treatment group only will also be explored: only imputations in the TAR group will be edited (missing data for the arthrodesis group remain imputed under MAR) and the Ø which causes the 52-week treatment effect estimate to cross the 0.05 boundary will be identified. Subsequently, only imputations for the arthrodesis group will be modified as described above. Alongside information on the characteristics of the patients missing 52-week MOXFQ scores, these analyses will be used to consider whether missing outcome data may compromise conclusions from the primary analysis.

## Discussion

This update contains the pre-specified statistical analysis plan for the TARVA trial, written to conform with the Journal of the American Medical Association Guidelines for the Content of Statistical Analysis Plans in Clinical Trials [28]. By publishing the statistical analysis plan we aim to increase the transparency of the data analysis. The TARVA trial will provide comprehensive and precise information on the relative effectiveness of TAR versus ankle arthrodesis.

## Supplementary information

**Additional file 1.** Dummy tables. This file contains dummy tables which show the planned format and contents of the tables for the TARVA final statistical report.

## Data Availability

The protocol has previously been published [6]. Following completion of the trial analysis the results will be published, and additional available data can be obtained by contacting the chief investigator (AJG). The study team retain exclusive use until publication of major outputs has been completed.

## Abbreviations

AE: Adverse event
AP: Anteroposterior
CT: Computed tomography
EQ-5D: EuroQol five-dimension quality-of-life instrument
FAAM: The Foot and Ankle Ability Measure
FAAM-ADL: The Foot and Ankle Ability Measure Activities of Daily Living subscale
FAAM-Sport: The Foot and Ankle Ability Measure Sport subscale
ICC: Intra-class correlation coefficient
ITT: Intention-to-treat; includes all observed data from randomised patients in accordance with their randomised procedure, regardless of whether surgery actually occurred
m: Average cluster size (patients per surgeon)
MAR: Missing-at-random
MCID: Minimal clinically important difference
MI: Multiple imputation
MNAR: Missing-not-at-random
MOXFQ: The Manchester-Oxford Foot Questionnaire
MRI: Magnetic resonance imaging
OA: Osteoarthritis
PIS: Patient information sheet
QoL: Quality of life
ROM: Range of motion
SAE: Serious adverse event
TAR: Total ankle replacement
TARVA: Total Ankle Replacement Versus Arthrodesis trial
T-score: A measurement of bone density expressed in standard deviation units from that of a healthy young adult
UCL CCTU: UCL Comprehensive Clinical Trials Unit
VAS: Visual analogue scale
