# Supplementary material for: A randomised, multi-centre trial of total ankle replacement versus ankle arthrodesis in the treatment of patients with end stage ankle osteoarthritis (TARVA): statistical analysis plan
Source: Trials. 2020 Feb 18;21:197. doi: 10.1186/s13063-019-3973-4 (PMC7029552; doi:10.1186/s13063-019-3973-4)
Supplement: Supplementary file 1 — Additional file 1. Dummy tables. This file contains dummy tables which show the planned format and contents of the tables for the TARVA final statistical report. [file 13063_2019_3973_MOESM1_ESM.docx]

# Additional File 1 – Dummy Tables

Table S1. Screening

| Reason for trial exclusion | N (%) |
| --- | --- |
| Ineligibility: Previous ipsilateral talonavicular, subtalar or calcaneocuboid fusion^*^ |  |
| More than 4 lower limb joints fused^**^ |  |
| Contraindication to both MRI and CT scan |  |
| History of local bone or joint infection |  |
| Co-morbidity^†^ |  |
| Participation in another clinical trial |  |
| Patient not interested: Strong treatment preference |  |
| No reason given |  |
| Other |  |

* or planned within 1 year of index procedure.

** including contralateral limb but excluding PIPJ fusions.

†any which in the opinion of the investigator is severe enough to interfere with the patient’s ability to complete the study assessments or present an unacceptable risk to the patient’s safety.

Table S2. Missing data in primary and secondary endpoints. Number (%) of patients

| Outcome | Baseline | | Week 26 | | Week 52 | |
| --- | --- | --- | --- | --- | --- | --- |
|  | TAR | Arthrodesis | TAR | Arthrodesis | TAR | Arthrodesis |
| MOXFQ standing/walking |  |  |  |  |  |  |
| MOXFQ pain |  |  |  |  |  |  |
| MOXFQ social interaction |  |  |  |  |  |  |
| EQ-5D Index |  |  |  |  |  |  |
| EQ-5D VAS |  |  |  |  |  |  |
| FAAM - ADL |  |  |  |  |  |  |
| FAAM - Sport |  |  |  |  |  |  |
| ROM dorsiflexion  ROM plantarflexion |  |  |  |  |  |  |

Table S3. Baseline Characteristics, n(%)

|  | TAR | Arthrodesis |
| --- | --- | --- |
| Age (years), mean (sd) |  |  |
| Gender: Male |  |  |
| Height (m), mean (sd) |  |  |
| Weight (kg), mean (sd) |  |  |
| Aetiology of OA: Post-traumatic |  |  |
| Primary |  |  |
| Rheumatoid arthritis |  |  |
| Other inflammatory |  |  |
| Other |  |  |
| OA in subtalar joint |  |  |
| OA in talonavicular joint |  |  |
| User of assistive devices |  |  |
| Assistive device: Crutches |  |  |
| Ankle brace |  |  |
| Frame |  |  |
| Wheelchair |  |  |
| Stick/cane |  |  |
| Wheeled walker |  |  |
| Knee scooter |  |  |
| Other |  |  |
| Taking Anti-coagulants |  |  |
| History of cancer |  |  |
| Chronic pain |  |  |
| Connective tissue disorder |  |  |
| Diabetes |  |  |
| Gastro-intestinal disease |  |  |
| Hypertension  Hypercholesterolaemia |  |  |
| Inflammatory disorder |  |  |
| Metabolic disorder |  |  |
| Neurological disorder |  |  |
| Obesity |  |  |
| Peripheral nervous system disorder |  |  |
| Peripheral vascular disease |  |  |
| Renal pathology |  |  |
| Current smoker |  |  |
| Cigarettes/day, mean (sd) |  |  |
| Ex-smoker  Time since cessation (years), mean(sd) |  |  |
| Thrombo-embolic disease |  |  |
| Other condition affective mobility |  |  |
| Patient preference: TAR |  |  |
| Ankle Arthrodesis |  |  |
| No preference |  |  |

Table S3. Baseline Characteristics (continued)

|  | TAR | Arthrodesis |
| --- | --- | --- |
| Fixed flexion deformity of the knee |  |  |
| Fixed equinus |  |  |
| Significant mal-alignment |  |  |
| ROM dorsiflexion (degrees), mean (sd)  ROM plantarflexion (degrees), mean (sd) |  |  |
| MOXFQ standing/walking, mean (sd) |  |  |
| MOXFQ pain mean (sd) |  |  |
| MOXFQ social interaction, mean (sd) |  |  |
| EQ-5D Index, mean (sd) |  |  |
| EQ-5D VAS, mean (sd) |  |  |
| FAAM – ADL, mean (sd) |  |  |
| FAAM – Sport, mean (sd) |  |  |

| Table S4. Outcome Measures at 26 and 52 weeks post randomisation | | |
| --- | --- | --- |
|  | TAR | Arthrodesis |
| *At 26 weeks, mean (sd)* |  |  |
| MOXFQ standing/walking |  |  |
| MOXFQ pain |  |  |
| MOXFQ social interaction |  |  |
| EQ-5D Index |  |  |
| EQ-5D VAS |  |  |
| FAAM-ADL |  |  |
| FAAM-Sport |  |  |
| *At 52 weeks, mean (sd)* |  |  |
| MOXFQ standing/walking |  |  |
| MOXFQ pain |  |  |
| MOXFQ social interaction |  |  |
| EQ-5D Index |  |  |
| EQ-5D VAS |  |  |
| FAAM-ADL |  |  |
| FAAM-Sport |  |  |
| ROM dorsiflexion (degrees) |  |  |
| ROM plantarflexion (degrees) |  |  |

| \| Table S5. Change in outcome measures from date of randomisation \| \| \| \| \| \| --- \| --- \| --- \| --- \| --- \| \|  \| TAR¹ \| Arthrodesis¹ \| Difference² [95% CI] \| P value \| \| *Change at 26 weeks, mean (sd)* \| \| \| \| \| \| MOXFQ standing/walking \|  \|  \|  \|  \| \| MOXFQ pain \|  \|  \|  \|  \| \| MOXFQ social interaction \|  \|  \|  \|  \| \| EQ-5D Index \|  \|  \|  \|  \| \| EQ-5D VAS \|  \|  \|  \|  \| \| FAAM-ADL \|  \|  \|  \|  \| \| FAAM-Sport \|  \|  \|  \|  \| \| *Change at 52 weeks, mean (sd)* \| \| \| \| \| \| **MOXFQ standing/walking *** \|  \|  \|  \|  \| \| MOXFQ pain \|  \|  \|  \|  \| \| MOXFQ social interaction \|  \|  \|  \|  \| \| EQ-5D Index \|  \|  \|  \|  \| \| EQ-5D VAS \|  \|  \|  \|  \| \| FAAM-ADL \|  \|  \|  \|  \| \| FAAM-Sport \|  \|  \|  \|  \| \| ROM dorsiflexion (degrees) \|  \|  \|  \|  \| \| ROM plantarflexion (degrees) \|  \|  \|  \|  \| \| ¹ Unadjusted change over time \| \| \| \| \| \| ² Difference in outcome at 26/52 weeks between treatments, adjusting for baseline values and OA in adjacent joints \| \| \| \|  \| \| *** Trial primary outcome** \| \| \| \| \| |
| --- | --- | --- | --- | --- | --- | --- | --- | --- | --- | --- | --- | --- | --- | --- | --- | --- | --- | --- | --- | --- | --- | --- | --- | --- | --- | --- | --- | --- | --- | --- | --- | --- | --- | --- | --- | --- | --- | --- | --- | --- | --- | --- | --- | --- | --- | --- | --- | --- | --- | --- | --- | --- | --- | --- | --- | --- | --- | --- | --- | --- | --- | --- | --- | --- | --- | --- | --- | --- | --- | --- | --- | --- | --- | --- | --- | --- | --- | --- | --- | --- | --- | --- | --- | --- | --- | --- | --- | --- | --- | --- | --- | --- | --- | --- | --- | --- | --- | --- | --- | --- | --- | --- | --- | --- | --- | --- | --- | --- | --- | --- | --- | --- | --- | --- | --- |

Table S6. Safety Outcome Measures, n(%)

|  | TAR | Arthrodesis | Absolute Diff  [95% CI] | P-value | Relative Risk [95% CI] |
| --- | --- | --- | --- | --- | --- |
| ≥1 Adverse Event |  |  |  |  |  |
| ≥1 Serious Adverse Event |  |  |  |  |  |
| Complications (any) |  |  |  |  |  |
| Revision surgery |  |  |  |  |  |
| Reoperation (other than revision)  Surgical site infection |  |  |  |  |  |
